# A Combination of Glucagon-Like Peptide-1 Receptor Agonist and Dietary Intervention Could Be a Promising Approach for Obesity Treatment

**DOI:** 10.3389/fendo.2021.748477

**Published:** 2021-09-20

**Authors:** Chooi Yeng Lee

**Affiliations:** School of Pharmacy, Monash University Malaysia, Subang Jaya, Malaysia

**Keywords:** combination therapy, diet, glucagon-like peptide-1 agonist, gut-brain axis, obesity

## Introduction

Obesity continues to be a global health issue. According to the World Health Organization (WHO), its prevalence has increased almost 3-fold between 1975 and 2016; thirteen percent of the adult population worldwide was obese in 2016 (1). The real concern behind the increasing number of obese individuals is that obesity is a risk factor of many debilitating diseases (1). Obesity was also found to worsen the effects of COVID-19 (2), the pandemic that has cost millions of lives globally (3). Obesity increases the risk of hospitalization and death among COVID-19 patients (4). It is therefore never ‘enough’ to discuss about existing strategies used in the management of obesity, more so when there are newer findings that could enlighten us on the underlying pathophysiology of obesity, thereby offering us new perspectives of the role of these approaches. This article focuses on glucagon-like peptide-1 (GLP1) - revisiting the physiological effects of GLP1, and proposing ways of optimizing the effects of GLP1 in the management of obesity.

## What Do We Already Knew About GLP1?

Glucagon-like peptide-1 (GLP1) is a peptide hormone produced by the L cells of the small intestine when the cells detected food. By binding to its receptor on the pancreatic β-cells, GLP1 stimulates insulin secretion in a glucose-dependent manner. GLP1 also exerts the following effects: suppresses pancreatic glucagon release as well as hepatic glucose production, promotes the proliferation of pancreatic β-cells, prevents apoptosis of β-cells, and delays gastric emptying (5). These physiological effects of GLP1 have led to the extensive development of GLP1-based anti-diabetic drugs. Interestingly, not only did GLP1 lower the hemoglobin A1C, subcutaneous GLP1 infusion reduces the body weight and appetite of diabetic patients (6). Zander and team were probably the first to report the presence of an inverse relationship between GLP1 and body weight and appetite, which has then sparked the interest of using GLP1 as an anti-obesity agent. Their findings were supported by studies conducted by other researchers subsequently. A review published a decade later (7) confirmed that GLP1 secretion is reduced in obese subjects, while weight loss normalizes the levels, and GLP1 replacement restores satiety.

Currently, Liraglutide is the only FDA-approved GLP1 receptor agonist for weight loss treatment in non-diabetic patients. Semaglutide was added to the list by FDA recently for chronic weight management in obese or overweight adults with at least one weight-related condition such as hypertension, diabetes, or hypercholesterolemia (8). However, the most effective clinical procedure that has resulted in significant long-term weight loss is bariatric surgery (9). Obese patients who underwent bariatric surgery have elevated levels of GLP1 and diminished appetite (10). The increased GLP1 may be one of the main factors that has contributed to the weight loss effect of bariatric surgery (11), although the metabolic improvements post-bariatric surgery are more likely to be contributed by several mechanisms (12). But as with other surgeries, complications may develop, and not all obese patients are suitable to undergo bariatric surgery. Taken into consideration the cost and safety, therapeutic agent such as GLP1 receptor agonist may be preferred over bariatric surgery as a treatment option, especially in non-morbidly obese patients. The effects of GLP1 receptor agonist can be optimized through our understanding of the pathophysiology of obesity as well as the effect of obesity on GLP1 production.

## What Is New and Not So New About Obesity Pathophysiology and Its Impact on GLP1?

A cohort study reported that individuals who are obese and overweight had a 20% reduction in the GLP1 response to oral glucose compared with subjects who have normal weight (13). While these results were recently debated by some researchers based upon their findings from animal studies (14), the causal-effect relationship remains possible, as discussed below. Variation in the effect of glucose response observed by different group of researchers can be attributed to the differences in the experimental environment, the animal strain used, and the experimental design. The physiological effect of GLP1 is not limited to insulin secretion, but a range of other effects, as mentioned above. One of the most important beneficial effects of GLP1 in obesity treatment is promoting satiety. This is because increased access to tasty, low-cost and energy-dense food is partially responsible for the increase in obesity prevalence (15, 16).

The appetite-suppressant effect of GLP1 is well known. Briefly, two sets of neuronal circuitry are present within the arcuate nucleus (ARC) of the hypothalamus. These neuronal circuits signal to the paraventricular nucleus and modulate feeding behavior. The neuronal circuit that expresses neuropeptide Y (NPY) and agouti-related peptide (AgRP) stimulates food intake, while the ARC neuron that expresses pro-opiomelanocortin (POMC) and cocaine- and amphetamine-regulated transcript (CART) reduces food intake. GLP1 suppresses the expression of NPY and AgRP, while increases the activity of POMC and CART (17). Besides GLP1 receptor agonists, another class of drug that has gained increasing interest for having an effect on appetite is the cannabinoid receptor type 1 (CB1R) antagonists. Peripherally-acting CB1R antagonists effectively reduced the body weight and appetite of diet-induced obesity (DIO) mice while improving the metabolic dysfunction of the animal (18). The insulinotropic effect of GLP1 was enhanced in CB1R-deficient mice (19). The combination of peripherally-acting CB1R antagonist and GLP1 agonist, Semaglutide was significantly more effective than CB1R antagonist alone or Semaglutide alone in lowering the body weight of DIO mice (20). The weight reduction effect of the CB1R antagonist was negated when GLP1 receptor was absent, while CB1R antagonist potentiated the weight loss effect of Semaglutide and improved Semaglutide’s response to glucose. The combination of CB1R antagonist, Rimonabant and Semaglutide also reduced food intake to a greater extent than monotherapies but this effect is not dose-dependent (20). The amplified anorectic effect of GLP1 by CB1R antagonist may be due to the fact that both receptors are expressed on the vagal afferent neurons, enabling a cross talk between the two receptors (21).

The concept that gut plays a critical role in obesity has been widely described. Most of these studies involved rodent treated with high fat diet (HFD), as the DIO model presents metabolic status that is closest to the human condition. Two months of HFD feeding increases the sensitivity of the gut to luminal nutrients, but the increased luminal sensitivity may not have stimulated GLP1 secretion (22). The possibility of HFD induces GLP1 secretion was not confirmed because the same group of researchers did not see a significant increase in GLP1 level in the distal small intestine where GLP1 is abundantly produced (23). In contrast to these findings, a few studies involving chronic HFD feeding (2-4 months) in rodent reported a decrease in GLP1 secretion in response to oral glucose (24–26). Several studies on the other hand showed that HFD terminates the gut lipid sensing mechanisms (27). For example, HFD diminishes the effect of cholecystokinin-8 (28) and long chain fatty acyl-coenzyme A (29) in reducing the hepatic glucose production. HFD also suppresses peroxisome proliferator-activated receptor-α (30) and N-acylphosphatidylethanolamine secretion (31) resulting in increased hunger. Collectively, it seems that HFD may increase luminal sensitivity but this effect is likely to adversely affect the metabolic status of obese subjects. It is more likely that the HFD-induced GLP1 secretion observed by some researchers was short-term or intermittent because HFD impaired the secretory function of the L cells (32), and GLP1 inversely correlates with appetite and body weight gain.

Human studies reported the presence of inflammatory proteins in the systemic circulation of obese patients, which correlates positively with body weight (33, 34). The inflammation observed in obese human subjects was supported by animal studies, which indicated that inflammation occurred during the course of obesity development (35). The interaction between HFD and gut microbiota triggers the inflammatory cascade causing intestinal inflammation. This effect was not found in germ-free mice (35), which were also resistant to DIO (36). By acting through G protein-coupled receptor 41 (Gpr41), microbiota increase adiposity and leptin production. Conversely, Gpr41-deficient mice have a decrease in peptide YY secretion as well as hepatic lipogenesis (37). It is possible that the effect of microbiota on the Gpr expressed on the enteroendocrine cells, which contributes to adiposity is exaggerated by HFD. This is because Gpr are fat sensors that mediate gastrointestinal peptides secretion. In another study, mice fed with 14 weeks of HFD had a significant increase in body weight and gastrointestinal methanogens compared with chow-fed mice. In conjunction with the increased abundance of fecal methanogens, there was elevated circulating GLP1 level (38). However, the elevated GLP1 level was observed at 10 minutes after oral glucose and not thereafter suggesting that the increase is transient. An *in vitro* study conducted subsequently by the same group of researchers, shows that the increased GLP1 in the cell culture media as induced by methane, was not dose-dependent. It is therefore not clear if the enhanced GLP1 secretion in DIO mice (38), which was thought to be contributed by methane is sustainable, and if this effect would lead to a reduction in body weight as well as appetite eventually. Prolonging the *in vivo* study beyond 14 weeks and measuring relevant parameters could confirm the methane effect on GLP1 secretion. In short, what is clearer at the moment is that HFD induces changes in the gut microbiome, and that causes inflammation and adiposity.

## How Shall We Progress From Here?

Research over the past two decades has established that GLP1 acts along the gut-brain axis, producing anti-obesity effects. But obesity negatively influences the effect of GLP1 centrally and peripherally through the appetite-regulating centre and the gut, respectively. A treatment regime that targets on both axes instead of one axis may therefore be more beneficial. This could mean increasing the appetite-suppressant effect of GLP1 and increasing or keeping the intestinal production of GLP1, both of which are achieved by improving the systemic bioavailability of GLP1. The bioavailability of GLP1 can be improved by developing GLP1 receptor agonists, which are resistant to enzyme dipeptidyl peptidase IV degradation. This approach has been widely adopted and most of the agonists have been approved for use in diabetic treatment (39). Liraglutide and Semaglutide, as mentioned above have been approved for use as weight loss agents. What is next then? Maintaining gut health appeared essential if one were to benefit from the physiological effects of GLP1, and ‘fend off’ the masking effects of DIO on GLP1 or the wide-ranging adverse effects of HFD or the impact of obesity on the gut.

## Effects of Dietary Interventions on the Gut and Body Weight

Many researchers have turned to dietary intervention, hoping that the approach would increase GLP1 secretion. Highly soluble and fermentable fibers, through the production of short chain fatty acids, promote GLP1 secretion (40). Fructooligosaccharides (41) and flavonoids (42) also acutely stimulated GLP1 secretion in rats. The use of oligosaccharides however may not be preferred as it may exacerbate irritable bowel syndrome, causing bloating, abdominal discomfort, and altering the movement of the intestine. Flavonoids consumption on the other hand should be encouraged. Gut antioxidants not only protect intestinal cells from inflammation, but also stress-induced body weight gain and food intake (43). Due to the hydrophilic nature of flavonoids, this plant-based antioxidant is poorly absorbed, and accumulated in the stomach and intestinal lumen. As proposed before (22), dietary antioxidant is an ideal alternative to conventional drugs for obesity treatment, more so if it could increase GLP1 secretion. Separately, if we are able to identify the specific microbial strains that influence the synthesis of GLP1, ingesting prebiotics that modify the activity of those strains may also be useful in increasing GLP1 secretion.

Dietary strategies are effective in reducing body weight. The effect is prolonged if the strategies are combined with behavioral counseling and ongoing support (44). High protein diet and low carbohydrate diet were suggested to be more beneficial than other types of diet (44). A meta-analysis of randomized controlled trials comparing low carbohydrate diet with low fat diet showed that the former improved weight loss more significantly than low fat diet in studies that ranged from 8 weeks to 24 months in duration (45). Mediterranean diet has been widely discussed lately (46, 47). There is no consensus on the definition of Mediterranean diet but the diet generally includes high levels of low carbohydrate and low protein food, and moderate levels of red wine and dairy products. Consumption of Mediterranean diet was found to produce greater weight reduction compared with low fat diets at 2 years (48, 49). The mechanism of the effects of Mediterranean has not been well investigated but an 8 weeks randomized controlled trial showed that the diet changed the gut microbiome of healthy obese and overweight subjects, decreasing microbial species that is proinflammatory, and had reduced systemic inflammation of the subjects (46). Regardless of which dietary strategy is implemented, adherence to the diet is the key to the long-term success of weight management or obesity treatment (46, 47, 50).

**Figure 1:**
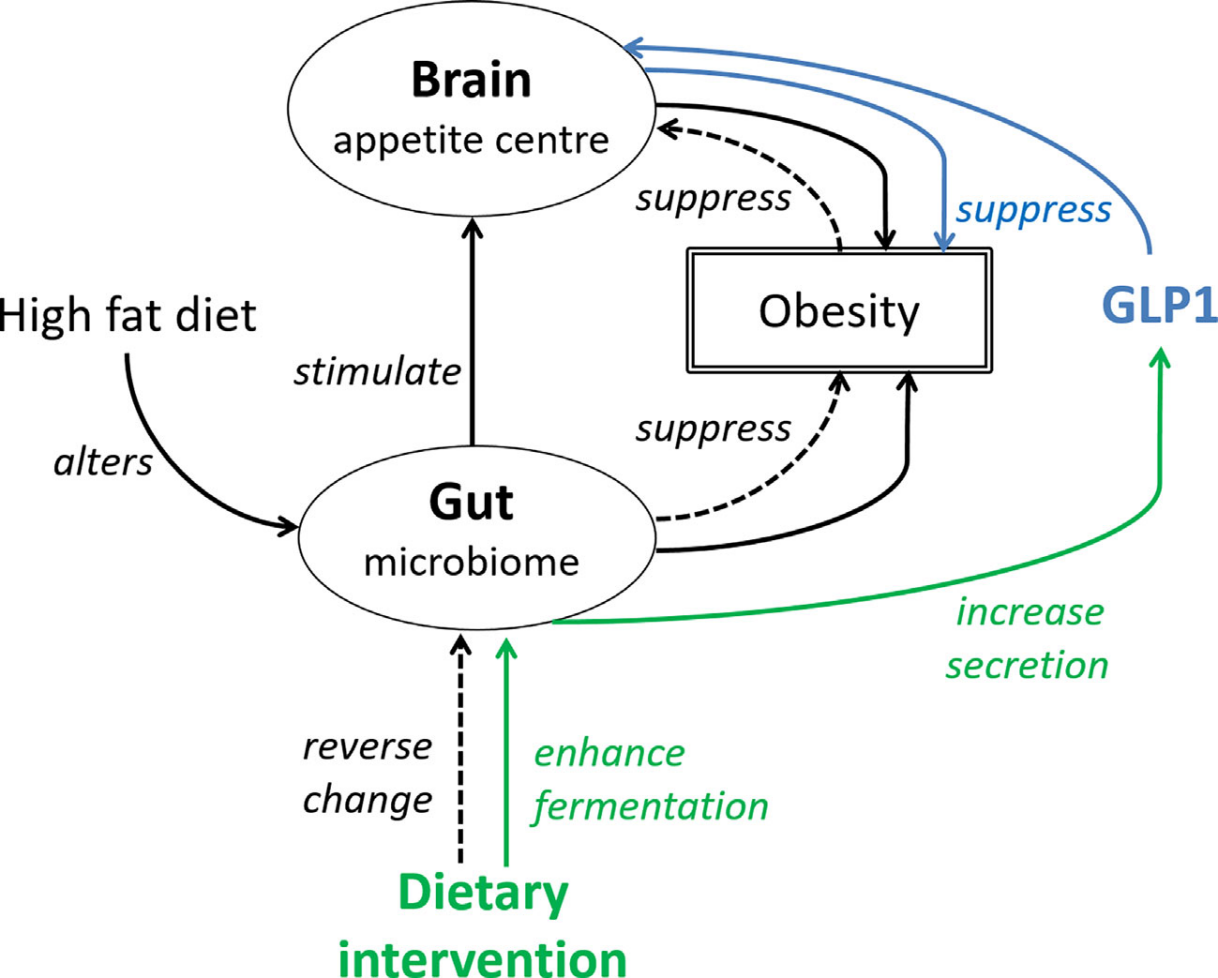
The gut-brain axis is central to the effects of GLP1. Solid black line: High fat diet alters the gut microbiome, which then stimulate the appetite leading to increased food intake and body weight gain. The interaction between HFD and gut microbiome also increases adiposity. Blue line (pharmacological approach): GLP1 or GLP1 receptor agonist suppresses appetite and reverses the increase in body weight. Green line (non-pharmacological approach): dietary fibers by enhancing fermentation in the large intestine, increase GLP1 secretion. Dotted line (non-pharmacological approach): dietary antioxidants may reverse changes of the gut microbiome induced by high fat diet, subsequently suppressing obesity and food intake.

Various plant-based food, which have weight and fat mass reduction effects (51, 52), and food not targeting at weight control or increasing GLP1 secretion but reducing inflammation, have been suggested (53). The latter approach is sensible because inflammation has been reported to contribute either to the progression or the manifestation of obesity. Overall, there is adequate evidence to show that dietary intervention is promising. A concerted effort between endocrinologist and dietitian may be more effective in improving the treatment outcomes of obesity than a single modality treatment. **Figure 1** gives an overview of the effect of HFD and gut microbiome on obesity development, and how targeting the gut-brain axis is beneficial.

## Conclusion

Currently available therapy has not been effective in curbing the upward trend of obesity prevalence, which should prompt us to reconsider the traditional approach of obesity treatment. The outcomes of various clinical trials suggest that adopting a holistic approach in weight management may be more effective than focusing on one specific strategy involving either drug or non-drug treatment. The holistic approach may comprise of a pharmacological therapy, implemented alongside a dietary intervention. Liraglutide and Semaglutide are promising weight reduction pharmacological agents. The treatment should be accompanied by behavioral therapy and on-going support for dietary changes. Long-term adherence to dietary intervention such as Mediterranean diet and low carbohydrate diet may help to produce a sustainable weight loss, while the consumption of food or herbal products with antioxidant and anti-inflammatory properties synergizes the effect of GLP1 receptor agonist and improves the overall health.

## Author Contributions

The author confirms being the sole contributor of this work and has approved it for publication.

## Conflict of Interest

The author declares that the research was conducted in the absence of any commercial or financial relationships that could be construed as a potential conflict of interest.

## Publisher’s Note

All claims expressed in this article are solely those of the authors and do not necessarily represent those of their affiliated organizations, or those of the publisher, the editors and the reviewers. Any product that may be evaluated in this article, or claim that may be made by its manufacturer, is not guaranteed or endorsed by the publisher.
